# Sleep-loss related to itch in atopic dermatitis: assessing content validity and psychometric properties of a patient-reported sleep-loss rating scale

**DOI:** 10.1186/s41687-024-00764-2

**Published:** 2024-07-22

**Authors:** Alissa Rams, Jessica Baldasaro, Laurine Bunod, Laure Delbecque, Sara Strzok, Juliette Meunier, Hany ElMaraghy, Luna Sun, Evangeline Pierce

**Affiliations:** 1 Modus Outcomes, a Division of THREAD, Cambridge, MA USA; 2 Modus Outcomes, a Division of THREAD, Lyon, France; 3 Eli Lilly and Company, Indianapolis, IN USA

**Keywords:** Atopic dermatitis, Itch, Mixed-methods research, Patient-reported outcome, Rating scale, Sleep impact

## Abstract

**Background:**

Sleep loss is a key factor contributing to disease burden in people with atopic dermatitis (AD). Mitigating itch to improve sleep is an important outcome of AD treatment. This study explored the content validity and measurement properties of the Sleep-Loss Scale, a single-item rating scale for assessing itch interference with sleep in clinical trials of AD treatments.

**Methods:**

Concept elicitation and cognitive debriefing interviews were conducted with 21 adults and adolescents (12–17 years of age) with moderate-to-severe AD to develop a conceptual model of patient experience in AD and explore the content validity of the scale. Data collected from adults with moderate-to-severe AD enrolled in a phase 2b study (NCT03443024) were used to assess Sleep-Loss Scale’s psychometric performance, including reliability, construct validity, and ability to detect change. Meaningful within-patient change (MWPC) thresholds were also determined using anchor-based methods.

**Results:**

Qualitative findings from concept elicitation highlighted the importance of sleep-loss related to itch in AD. Debriefing analysis of the Sleep-Loss Scale indicated that the scale was relevant, appropriate, and interpreted as intended. Trial data supported good reliability, construct validity and ability to detect improvement. MWPC was defined as a 1-point improvement using trial data, a finding supported by qualitative data.

**Conclusions:**

The Sleep-Loss Scale provides a valid and reliable patient-reported measure of the impact of itch on sleep in patients with AD, and can detect change, indicating it is fit-for-purpose to evaluate the efficacy of AD treatments in moderate-to-severe patients.

**Supplementary Information:**

The online version contains supplementary material available at 10.1186/s41687-024-00764-2.

## Key summary points

• The Sleep-Loss Scale is a novel rating scale for assessing the impact of itch on sleep in atopic dermatitis (AD).

• A mixed-methods approach was used to explore the content validity and psychometric properties of the Sleep-Loss Scale.

• Qualitative results supported sleep loss as a common experience in AD and indicated that the Sleep-Loss Scale was relevant, appropriate, and interpreted as intended.

• Quantitative results demonstrated the Sleep-Loss Scale is valid, reliable, and able to detect change.

• The Sleep-Loss Scale was found to be fit-for-purpose for use in clinical trials of AD treatments among moderate-to-severe patients.

**Supplementary Information:**

The online version contains supplementary material available at 10.1186/s41687-024-00764-2.

## Introduction

Atopic dermatitis (AD) is a chronic, inflammatory skin disease in which itching is a key, hallmark symptom [1]. One of the most common skin diseases, AD primarily appears in childhood, although it can present at any time; in fact, 26% of adults with AD report onset during adulthood [2]. People with AD often report physical and psychological distress related to their condition, and AD is associated with high burden of disease and decreased quality of life in both adults and children [3–5]. In fact, AD has the highest disease burden among skin diseases as measured by disability-adjusted life years [6].

Sleep loss is a key factor that contributes to both high disease burden and lower quality of life in children and adults with AD [7–11]. While other factors such as flaking and skin pain can contribute to sleep loss in AD, itching is generally considered the main factor leading to sleep problems in AD patients [9]. As such, mitigating itch and its impact on sleep is an important outcome in AD treatment. Treatment for AD varies based on disease severity, but generally includes the use of topical anti-inflammatory medications (e.g., corticosteroids). Patients who do not respond to topical treatments may require systemic treatment [2]. Disease management can be difficult due to the heterogeneity of the disease; therefore, understanding how patients are affected by the disease is essential to provide them with appropriate resources.

Patient-reported outcome measures (PROs) are recommended by the US Food and Drug Administration (FDA) to measure aspects of a disease and its treatment that are important to patients. This is particularly important in cases where components of the disease experience (e.g., itch and its impact on sleep) are non-observable and known only to the patient [12]. However, in AD, there is no gold-standard PRO that enables patients to report on itch interference with sleep.

A recent Phase 2b study assessing the efficacy and safety of lebrikizumab in adult patients with moderate-to-severe AD used a simple, novel PRO instrument, the Sleep-Loss Scale, to measure the interference of itching on patients’ sleep [13]. The purpose of this mixed-methods study was to gather evidence regarding the content validity and measurement properties of the Sleep-Loss Scale, in order to determine whether this novel PRO is fit-for-purpose [14] to measure the experience of sleep-loss related to itch in adults and adolescents with moderate-to-severe AD in clinical trials.

## Methods

**Fig. 1:**
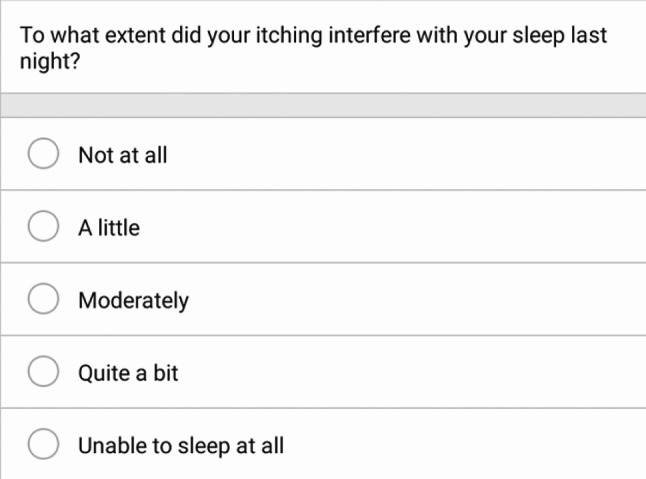
Sleep-Loss Scale

This mixed methods study regarding the Sleep-Loss Scale included two components: a qualitative component with adult and adolescent patients with AD and quantitative component involving psychometric analysis of Phase 2b clinical trial data (NCT04250350). The Sleep-Loss Scale is presented in Fig. 1.

### Qualitative methods

The qualitative component of this study was a non-interventional, descriptive, cross-sectional study comprising one wave of patient interviews using a 1:1 interviewing approach.

Patients eligible to participate in the study were 12 years of age or older with a clinician-confirmed diagnosis or symptoms of AD for at least one year prior to interview. Additional inclusion criteria included a history of inadequate clinical response to at least one treatment and ≥ 10% body surface area (BSA) of AD involvement within the past 45 days. Participants were also required to have access to the internet and the ability to view patient-facing materials in an online format other than a smartphone (e.g., tablet or computer). Patients with concomitant illness that would influence study assessments or who had received treatment with biologic treatments prior to screening for the study were excluded.

Study documents, including the protocol, demographic and health information form, interview guide, screener, and informed consent and assent forms received ethical approval from WCGIRB (IRB# 1298154). All participants provided informed consent/assent to participate in this study; adolescent participants provided assent, and their parent or guardian provided consent.

Study personnel conducted the interviews using a semi-structured interview guide. All interviewers attended specific training to review the objectives of the interviews and to address any questions regarding the interview guide. Virtual interviews were conducted in English and lasted approximately 60 min. Interviews were audio-recorded, transcribed verbatim, and anonymized. De-identified transcripts were used for qualitative analysis.

#### Concept elicitation

Concept elicitation was conducted to explore AD symptoms and their impact on sleep, as well as daily life impact of AD-related sleep loss, using open-ended questions. Participants were encouraged to describe in their own words their experience, severity, and fluctuation of the concepts of interest. Targeted probes were used to obtain specific information on concepts of interest after participants had the opportunity to respond to the open-ended questions spontaneously.

#### Cognitive debriefing

Cognitive debriefing of the Sleep-Loss Scale was conducted with participants to elicit their opinions on how well it covered the experience of sleep-loss related to AD. Participants completed the Sleep-Loss Scale online via REDCap, a secure, web-based application developed to capture data for clinical research [15]. A think-aloud process with specific probes tailored to the study’s objectives was used [16–21] to review the item text, response scales, and instructions. The overall presentation template of the PRO instrument (i.e., a screenshot of the electronic mode of administration used in the clinical trials), was also shared with participants on a subsequent survey page in REDCap so that participants could share their impressions of the format.

#### Qualitative analysis

Interview transcripts were analyzed thematically [22] through detailed line-by-line open and inductive coding [23, 24] using ATLAS.ti software. Independent parallel coding was used to initiate the coding and ensure consistency using the first two interviews (AR, JB, SS). The coding was revised as needed to reach coder consensus and following the identification of new concepts in the remaining transcripts, with iterative revisions to the codebook.

Codes were organized to illustrate concepts of interest, i.e., the experience of AD-related sleep loss from the patient perspective. The coding was targeted to itch-related sleep loss and its impacts. Broader concepts related to other AD symptoms and impact were also coded to understand patients’ experience of sleep interference in the context of these symptoms and to build a more comprehensive conceptual model of disease experience. The cognitive debriefing analysis identified and categorized feedback on the Sleep-Loss Scale spontaneously reported by participants and specifically probed by the interviewers.

### Quantitative methods

#### Data collection

The clinical data used in this study were from a phase 2b, randomized, double-blind, placebo-controlled, parallel-group, 16-week clinical trial that aimed to evaluate the efficacy, safety, and dose-response of lebrikizumab in patients with moderate-to-severe AD (NCT04250350) [13].

All patients completed the Sleep-Loss Scale, which consists of a single question “To what extent did your itching interfere with your sleep last night?”. Response options range from 0 (“Not at all”) to 4 (“Unable to sleep at all”). The Sleep-Loss Scale was collected daily, and weekly mean scores were used as endpoints. For each visit, weekly mean Sleep-Loss Scale score was computed for the week preceding each clinic visit, regardless of the number of assessments.

Several additional measures were completed by both clinicians and patients during the trial. Clinicians completed the Investigator Global Assessment (IGA), Body Surface Area (BSA), and Eczema Area and Severity Index (EASI). Patients completed the Pruritus Numeric Rating scale (PNRS) which measures patient-reported itch severity, the Patient Oriented Eczema Measure (POEM) which assesses disease activity, the Dermatology Life Quality Index (DLQI) which assesses quality-of-life impact of dermatological conditions on patients, and Hospital Anxiety and Depression Scale (HADS) which assesses anxiety and depression. Further information regarding the measures and the timing of the assessments can be found in the supplementary material.

### Statistical analysis

All psychometric analyses were performed in the modified Intent-to-Treat (mITT) population (i.e., all participants who were randomized and received the study drug). As psychometric analyses are independent of the question of treatment received, all analyses were performed on pooled data, blinded from the treatment group. Missing PRO scores and other clinical assessments were not imputed.

Demographic and clinical characteristics of the sample were described. Weekly mean Sleep-Loss Scale scores were described at each visit, including number of missing scores. The psychometric analyses of the Sleep-Loss Scale were performed in the classical test theory (CTT) framework. CTT analyses evaluated reliability, construct validity, and the ability of the scale to detect change over time. Test-retest reliability coefficients were estimated using interclass correlation coefficients (ICCs) between baseline and Week 4 and between Week 12 and Week 16 in a subsample of stable participants between the two visits defined by the IGA. Construct validity was studied by computing polychoric correlations between the Sleep-Loss Scale score and other clinical outcome assessment scores. The distribution of the Sleep-Loss Scale scores was also examined across POEM and DLQI categories. Ability to detect change over time was evaluated by calculating Kazis’ effect sizes (ES) [25, 26] in categories of participants defined according to the change in IGA, the Global Assessment of Change—Atopic Dermatitis (GAC-AD) and the change in POEM score. ES’s were interpreted according to Cohen’s recommendations [27].

Meaningful within-patient change was explored at Week 16 using anchor-based methods [28–30] and distribution-based methods [31] with the change in IGA and GAC-AD as anchors. Anchor-based methods included the use of Receiver-Operating Characteristic (ROC) curves of the Sleep-Loss Scale score according to various dichotomizations of the GAC-AD and change in IGA, as well as mean Sleep-Loss Scale score according to the groups defined by the anchors.

Data analysis was performed using SAS software version 9.4.

## Results

### Qualitative results

Participant interviews were conducted between February and September 2021. Twenty-one people (*n* = 15 adult, *n* = 6 adolescent) participated and were between the ages of 12–64 years. All participants had moderate-to-severe AD and reported experiencing itch within the past 24 h. Participant demographic and clinical characteristics are summarized in Table 1.

**Table 1:** Qualitative AD study: adult and adolescent demographic and clinical characteristics

	Adults (*n* = 15)	Adolescents (*n* = 6)
Age (in years)		
Mean (SD)	30.4 (12.9)	13.0 (1.0)
Min-Max (years)	19–64	12–15
Gender, n (%)		
Female	11 (73%)	3 (50%)
Race, n (%)		
Asian	8 (53%)	5 (83%)
Black	0 (0%)	1 (17%)
Native Hawaiian/Pacific Islander	1 (7%)	0 (0%)
White	4 (27%)	0 (0%)
Biracial	1 (7%)	0 (0%)
Missing	1 (7%)	0 (0%)
Ethnicity, n (%)		
Non-Hispanic/Non-Latino	13 (87%)	6 (100%)
Education level, n (%)		
Elementary/primary school	0 (0%)	3 (50%)
Some high school	0 (0%)	3 (50%)
Some college	5 (33%)	0 (0%)
Associate degree	1 (7%)	0 (0%)
Bachelor’s degree	7 (47%)	0 (0%)
Post-graduate	1 (7%)	0 (0%)
Trade	1 (7%)	0 (0%)
Time since first symptom, n (%)		
1–5 years	4 (27%)	3 (50%)
6–10 years	11 (73%)	3 (50%)
Time since diagnosis, n (%)		
Less than 1 year	1 (7%)	0 (0%)
1–5 years	6 (40%)	3 (50%)
6–10 years	8 (53%)	3 (50%)
Medications, n (%)^a^		
Triamcinolone	3 (20%)	1 (17%)
Hydrocortisone	2 (13%)	1 (17%)
Tacrolimus	3 (20%)	0 (0%)
Crisaborole	4 (27%)	2 (34%)
Halobetasol	1 (7%)	0 (0%)
Over the counter	5 (33%)	2 (34%)
None	2 (13%)	1 (17%)
Overall health, n (%)		
Fair	3 (20%)	2 (34%)
Good	4 (27%)	3 (50%)
Very good	5 (33%)	1 (17%)
Excellent	2 (13%)	0 (0%)

^a^Does not sum to 100% as participants could report multiple medications

Most participants reported some degree of itch interference with sleep the previous night, with 9 patients (43%) reporting a little interference and 6 patients (29%) reporting moderate interference (Table 2). No participants reported the highest rating of interference (being unable to sleep at all).

**Table 2:** Patient-reported itch interference with sleep as assessed by the sleep-loss scale

Sleep-Loss Scale itch interference with sleep rating	Adult (*n* = 15)	Adolescent (*n* = 6)
Not at all	2 (13%)	0 (0%)
A little	5 (33%)	4 (66%)
Moderately	5 (33%)	1 (17%)
Quite a bit	3 (20%)	1 (17%)
Unable to sleep at all	0 (0%)	0 (0%)

#### Concept elicitation

**Fig. 2:**
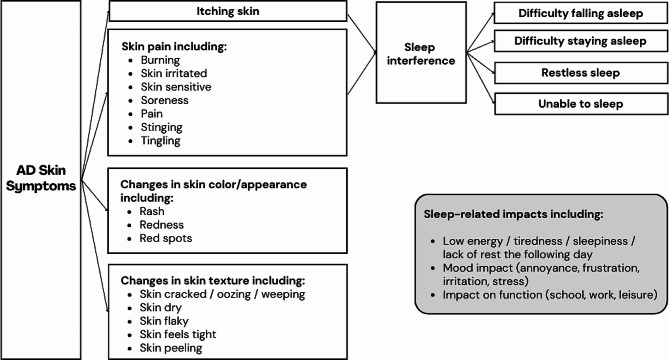
Conceptual model of AD symptoms and sleep interference

Twenty-two unique concepts related to AD symptoms and sleep impact and 41 unique concepts related to daily life impact were identified from the interview data. AD symptoms reported by participants included itch, pain, discomfort, dry skin, rash, redness, skin tightness, flakiness, soreness, and stinging. AD impacts reported by participants included impact on sleep, impact on daily life activities such as work, school, socializing, exercise, and household tasks, and emotional impacts such as annoyance, anxiety, frustration, depression, stress, and feeling stigmatized by the appearance of skin and the need to scratch. Aside from sleep loss attributed to itch, daily life changes made to mitigate AD symptoms (e.g., wearing different types of clothing, avoiding symptom triggers such as hot temperatures) were the most frequently reported impacts in this sample. There were no meaningful differences in concepts reported by adults vs. adolescents. Conceptual saturation was met for symptoms related to AD, but not for impacts. A conceptual model was developed to represent participants’ experience of sleep interference, itching, and other skin symptoms (Fig. 2).

#### Sleep interference

All patients reported that they had experienced sleep interference due to AD symptoms at some point over the course of the disease (*n* = 12 probed, *n* = 9 spontaneously). Most patients (17/21) explicitly linked problems with sleep to itching during the concept elicitation section of the interview. Patients stated that interference with sleep included taking longer to fall asleep, frequent waking over the course of the night, and not being able to sleep at all. Impacts of sleep interruption included frustration, annoyance, impact on other family members, and not feeling rested even after spending an adequate amount of time asleep, which was associated with decreased energy the following day. Table 3 summarizes patient reflections concerning sleep interference in AD.

**Table 3:** Sleep interference from itch in AD: exemplary patient quotes

Sleep interference	Patient statements
Difficulty falling asleep	“I don’t want to go to sleep because I know I’m scared that I’m going to move in a way that I’m going to get really itchy. I get really nervous and angsty at night because I know that’s when I get my worst type of itch problem.”– 01–007
	“I can’t sleep some nights. Some of those nights, it gets a little much, and I have trouble fully falling asleep from itchiness. Some nights are just really bad.”– 01–016
Difficulty staying asleep	“…even when I’m sleeping, I sometimes itch. Like sometimes it either keeps me up awake, or I wake up to many either rashes or open wounds on my body.”– 01-014a
	“It affects my sleep in that there are times where I have to take Benadryl to sleep so that I won’t wake up and find myself scratching. It’s an interrupted sleep because I do wake up in the middle of the night and feel the need to scratch… So interrupted sleep is huge. It doesn’t necessarily affect me falling asleep, but to stay asleep all night is definitely a huge problem, which then affects your energy the next day.”– 02 − 001
	“So I guess it affected me more when I was younger. I guess the itching was just so unbearable that I would wake up in the middle of the night having to scratch my body, and it just was so bad that one of my parents would have to sleep with me. And I would feel bad, but I would wake them up in the middle of the night for them to scratch me. And that– it lasted maybe for– I want to say two to three years.”– 01–027
Restless sleep	“I’m sure, at some points, there’s probably a lot of times that I’m not coherent of being disturbed by it, where you kind of do a little bit of a wake up but then go back into a deeper sleep, but in the morning you don’t remember it– kind of like with my roommate saying I said ow. I’m assuming that happens quite often, because I don’t always feel super rested after even eight, nine hours of sleep.”– 01–005
Unable to sleep	“Some nights I just can’t sleep at all because I just– or I’ll just wake up in the middle of the night and I just feel uncomfortable. My skin is just itchy.”– 01–004
	“You just feel the itching at all times, and you just can’t sleep because you’re constantly irritated by it.”– 01-018a
Impact of sleep loss on following day	“And that’s pretty common for me, even after eight, nine hours of sleep, to wake up still tired. And I’m not saying like just that first wakeup grogginess that most people have. I mean it’s persistent throughout the day. I don’t just wake up an hour later fully awake, my brain now awake, and I feel better. It’s like a consistency throughout my day.”– 01–005

#### Cognitive interviews

Cognitive interview results indicated that the Sleep-Loss Scale is relevant, appropriate, and interpreted as intended by adults and adolescents. Findings indicated that respondents could distinguish between itch-related sleep interference and other factors that cause sleep interference. The recall period and response scale were acceptable and well-understood, and patients were able to distinguish among the five severity levels of the response scale.

Nineteen participants rated their previous night’s sleep interference from 1—“a little” to 3—“quite a bit” on the Sleep-Loss Scale. No participants reported issues or difficulty with understanding the item as written. The word “interfere” was explicitly probed with adolescent participants, and all six were able to define “interfere” as getting in the way of or disrupting. The recall period was well-understood, with no participants misinterpreting the recall period.

The response scale was also well understood and easy to use: five participants explicitly endorsed the response scale as is, three indicated that the highest (“Unable to sleep at all,” *n* = 2) or lowest (“Not at all,” *n* = 1) response had not ever been part of their AD experience, and two stated that they thought that “a little” and “moderately” were similar to each other. Participants were able to distinguish between different levels on the response scale and all participants were able to select a response.

When selecting a response, participants considered itching interference with falling asleep, including how easy or difficult it was to fall asleep, how long it took to fall asleep, and the need to take proactive action to prevent itching before they went to sleep. Most patients (*n* = 11) said that a 1-point decrease in Sleep-Loss Scale score would indicate meaningful improvement; however, three patients stated that a 2-point change would indicate meaningful improvement. Patients described improvement on the Sleep-Loss Scale as changes to the sleep experience, including falling asleep more quickly, enjoying better quality sleep, feeling more rested on waking, functioning better at work or school and socially, and feeling less irritable or frustrated. Most patients (*n* = 12) indicated that a 2-point increase in Sleep-Loss Scale score would indicate meaningful worsening. No clear differences between adults and adolescents were detected in the cognitive debriefing analysis.

### Quantitative results

The mITT population of the phase 2b trial comprised 280 people, with a mean (standard deviation [SD]) age of 39 ± 17 years. The sample was predominantly female (59%) and white (52%). See Table 4 for additional demographic information. More extensive demographic and clinical information have been presented fully elsewhere [13].

**Table 4:** Study DRM06-AD01: demographic and medical information at baseline in the mITT* population

Variable	mITT Population (*N* = 280)
Age (in years)	
Mean (SD)	39.30 (17.48)
Gender, n (%)	
Female	166 (59.3%)
Race, n (%)	
American Indian or Alaska Native	3 (1.1%)
Asian	27 (9.6%)
Black or African American	93 (33.2%)
White	145 (51.8%)
Multiple	4 (1.4%)
Other	8 (2.9%)
Ethnicity, n (%)	
Hispanic or Latino	42 (15.0%)
Years with atopic dermatitis	
n	279
Mean (SD)	23.06 (16.59)

*mITT modified Intent-to-Treat, *SD* standard deviation

Most participants (65%) had moderate AD and 35% had severe AD, according to the IGA. Participants had severe eczema according to the POEM scores (mean ± SD = 20 ± 6), and AD had a very large effect on patient quality of life according to the DLQI scores (mean ± SD = 14 ± 7). The baseline PROs and clinician-reported outcomes (ClinROs) are located in Table 5.

**Table 5:** Description of PRO and ClinRO data at baseline in the mITT population

Variable	mITT Population	
	Baseline (*N* = 280)	Week 16 (*N* = 202)
Sleep-Loss Scale score		
n	262	180
Mean (SD)	2.04 (1.13)	0.86 (0.97)
Median	2.00	0.57
Min, Max	0.00, 4.00	0.00, 3.57
PNRS score		
n	261	180
Mean (SD)	7.46 (2.18)	3.62 (2.88)
Median	8.00	3.00
Min, Max	0.00, 10.00	0.00, 10.00
POEM-Score		
n	279	202
Mean (SD)	20.35 (6.21)	10.18 (7.11)
DLQI-Score		
n	279	202
Mean (SD)	14.22 (7.18)	5.44 (5.54)
HADS- Anxiety score		
n	280	202
Mean (SD)	7.50 (4.70)	5.37 (4.69)
HADS- Depression score		
n	280	200
Mean (SD)	5.52 (4.21)	3.31 (3.65)
IGA Score, n (%)		
Clear	0 (0.0%)	21 (10.4%)
Almost Clear	0 (0.0%)	50 (24.8%)
Mild	0 (0.0%)	63 (31.2%)
Moderate	182 (65.0%)	57 (28.2%)
Severe	98 (35.0%)	11 (5.45%)
Body Surface Area		
n	280	202
Mean (SD)	42.79 (22.43)	20.34 (23.04)
EASI Score		
n	280	202
Mean (SD)	27.41 (11.75)	9.22 (11.15)

*mITT* modified Intent-to-Treat, *SD* standard deviation, *PNRS* Pruritus Numeric Rating Scale, *POEM* Patient Oriented Eczema Measure, *DLQI* Dermatology Life Quality Index, *HADS* Hospital Anxiety and Depression Scale, *IGA* Investigator’s Global Assessment, *EASI* Eczema Area and Severity Index

The weekly mean Sleep-Loss Scale score was missing for less than 10% of the participants at each visit, ranging from 3.1% of missing weekly Sleep-Loss Scale score at Week 4 to 7.1% at Week 12, except at Week 14 (10.1% of missing weekly Sleep-Loss Scale score) and at Week 16 (10.9%), based on the number of participants still in the study at each visit.

In stable participants (as defined by no change in IGA score), ICC was 0.39 between baseline and Week 4 (*n* = 107), and 0.81 between Week 12 and Week 16 (*n* = 109).

Table 6 describes the correlations between the Sleep-Loss Scale and the IGA, EASI, BSA, DLQI, POEM, and HADS scores at baseline and Week 16 in the mITT population. Lower correlations were observed with clinical outcomes than with PROs, aside from HADS scores. Correlations were higher at Week 16 than at baseline, with the highest correlation found with the POEM sleep item (0.9 at Week 16).

**Table 6:** Correlation between the sleep-loss scale and IGA, EASI, BSA, DLQI, POEM, and HADS at baseline and week 16 in the mITT population

	Polychoric correlation coefficients	
	Sleep-Loss Scale score at baseline *N* = 262	Sleep-Loss Scale score at Week 16 *N* = 180
IGA Score	0.09	0.52
EASI Score	0.24	0.48
Body Surface Area	0.17	0.48
DLQI-Score	0.57	0.66
POEM-Score	0.55	0.66
POEMQ02-Nights of Disturbed Sleep	0.69	0.90
HADS- Anxiety score	0.26	0.18
HADS- Depression score	0.32	0.16

Low correlations: *r* < 0.30; moderate correlations: 0.30 ≤ *r* < 0.70; high correlations: *r* ≥ 0.70

**Fig. 3:**
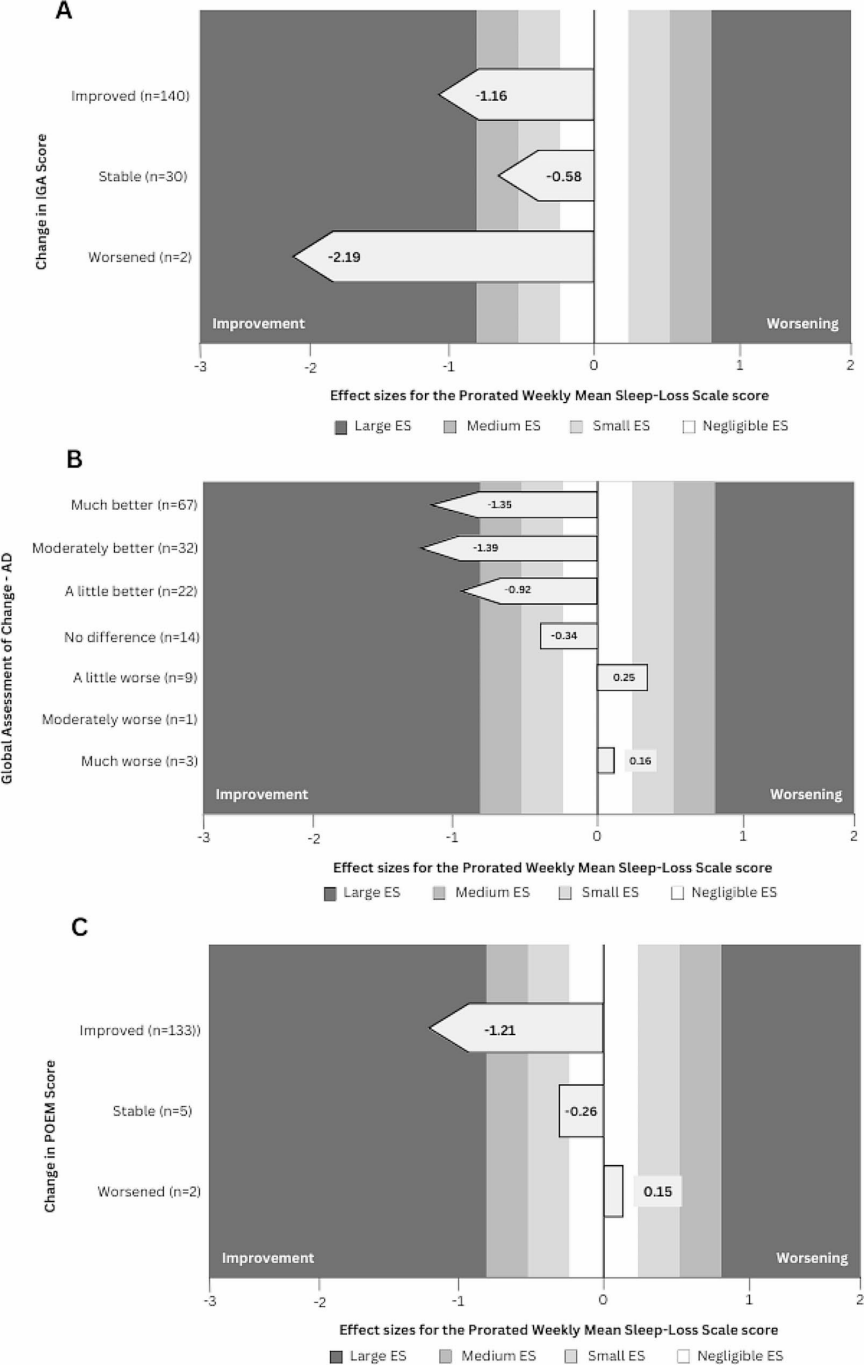
Effect sizes of the Sleep-Loss Scale according to the change in IGA (**A**), the GAC-AD (**B**), and the change in POEM score (**C**)

Large ESs (> 0.80) were observed for improvement at Week 16 according to the change in IGA (ES = −1.16, see Fig. 3A), according to the GAC-AD (ES = −1.35 for participants categorized as “Much better”, ES = −1.39 for participants categorized as “Moderately better” and ES = −0.92 for participants categorized as “A little better”, see Fig. 3B), and according to the change in POEM score (ES = −1.21, see Fig. 3C). Since very few participants were classified as worsened, no conclusions were drawn for the ES for worsened participants.

The correlation of the change in weekly mean Sleep-Loss Scale score from baseline to Week 16 was 0.42 with the GAC-AD and 0.27 with the change in IGA. Change in IGA was not correlated sufficiently (< 0.30) with the Sleep-Loss Scale to be used as a strong anchor for the meaningful change analyses of the Sleep-Loss Scale.

Based on a qualitative assessment of results from anchor- and distribution-based methods (see Table 7), the suggested value for meaningful within-individual improvement for the Sleep-Loss Scale was − 1.

**Table 7:** Results of anchor- and distribution-based methods

Anchor	Method	Thresholds based on anchor dichotomizations				Distribution-based range
		Improvement vs. all others	Improvement vs. no change	Moderate improvement vs. no change	Minimal improvement vs. no change	
GAC-AD	ROC	−1.00	−1.00	−1.00	0.00	0.49–0.88
	MEANS	−1.24	−0.94	−0.99	−0.34	
Change in IGA	ROC	−1.00	−1.00	−1.00	−1.00	
	MEANS	−0.75	−0.77	−0.72	−0.65	

*GAC-AD* Global Assessment of change—Atopic Dermatitis, *IGA* Investigator Global Assessment

As the Sleep-Loss Scale is a five-point scale, no range was suggested for meaningful within-individual improvement.

## Discussion

Overall, the results of this mixed-methods study highlight the experience of sleep loss due to itch as a significant part of the AD experience. This study also indicates that this experience can be captured by the Sleep-Loss Scale in clinical settings.

In qualitative interviews, sleep interference was reported as an important impact of AD, with all participants reporting that they had experienced sleep interference due to AD symptoms at some point over the course of the disease. Most participants explicitly linked problems with sleep to itching and described several types of sleep interference related to itch, including difficulty falling asleep, difficulty staying asleep or waking up at night, restless sleep, and inability to sleep at all. People with AD also indicated that their ability to function and interact was negatively impacted following a night of sleep interference due to itch. These findings align with existing literature, which has shown that itch is a main factor leading to sleep problems among patients with AD [9].

Results from cognitive interviews indicated that the single Sleep-Loss Scale item captures a core aspect of AD in a way that is commonly experienced by patients. Patients understood the 5-point response scale and could select a response that matched their experience. The debriefing also highlighted that patients specifically considered the impact of itch upon sleep, rather than other factors, when responding to the item. Importantly, no clear differences appeared in cognitive debriefing results between adults and adolescents, indicating that this instrument is appropriate for both age groups.

Results from the psychometric analyses of phase 2b study data showed that the Sleep-Loss Scale had strong measurement properties in this context of use. The scale showed good test-retest reliability (ICCs > 0.8) between Week 12 and Week 16 in the sample of stable patients defined by the change in IGA (i.e., patients with no change in IGA between Week 12 and Week 16) [32]. While test-retest reliability was poor between baseline and Week 4 in the subsample of stable patients, the IGA was poorly correlated with the Sleep-Loss Scale at the beginning of the study, explaining this result. Patients and clinicians may not have the same perception of the impact of itch on sleep at the very beginning of the study. Patients’ and clinicians’ perceptions of the concepts measured by the IGA and the Sleep-Loss Scale may also change at different rates, especially given the single-item Sleep-Loss Scale’s sensitivity to change.

This difference between patient and clinician perspectives was illustrated further through construct validity analyses: correlations between the Sleep Loss Scale and clinical assessments were negligible to low at baseline and moderate to high at Week 16, showing a better agreement between patients’ and investigators’ perceptions of the disease at the end of the study. A potential hypothesis is that treatment benefit may be more visible at the end of the study and thus noticeable by clinicians, while patients may perceive a non-visible benefit of the treatment from the very beginning of the study. Patient and clinician assessments of disease severity often differ [33]; in this case, patients may have perceived the benefit of the AD treatment before it was apparent from skin appearance, especially given that itch and its impact on sleep are non-observable and known only to the patient.

The Sleep-Loss Scale demonstrated a good ability to detect improvement over time. Large ESs (>|0.8|) were observed for improvement according to the change in IGA, GAC-AD, and change in POEM scores. It was not possible to conclude on the ability to detect worsening of the scales as there were too few worsened patients. The quantitative study confirmed the 1-point change as a meaningful improvement in the Sleep-Loss Scale as reported by participants of the qualitative study. Specifically, participants stated that meaningful improvement or a −1 point within-individual change on the Sleep-Loss Scale would indicate that they were able to fall asleep and stay asleep without interference from itching, and that improved sleep would result in improved mood and allow them to function better at work and school.

Some limitations of this study should be acknowledged. Targets were not met for sex at birth (33% male rather than 40%); however, the prevalence of AD is slightly higher in females than in males [34, 35]. More importantly, though this study sample was somewhat racially diverse, the majority of patients (*n* = 13) were Asian; only four white and one Black participant were included in the study, which may limit the generalizability of results, especially when considering the higher rates of AD in Black US population [34, 35]. Though the study did not meet the targets for the total number of participants (*n* = 30) and for participants aged ≤ 17 in particular, the results of conceptual saturation analysis indicated that conceptual saturation was achieved. Finally, the design of the clinical trial was not meant to assess the psychometric properties of the Sleep-Loss Scale specifically; for example, the time between two assessments (baseline and week 4) for the assessment of the test-retest reliability may have been too long, and thus the psychometric properties of this scale need to be confirmed in other studies. They should also be confirmed in an adolescent population as only adults were included in the phase 2b trial.

## Conclusion

Findings from this study add to the body of literature indicating that sleep loss due to itch is a relevant and meaningful concept for AD patients. The Sleep-Loss Scale is a valid and reliable tool that can detect change in itch-related sleep loss. A 1-point improvement on the scale reflects a meaningful within-person change, according to qualitative and quantitative data. Therefore, the Sleep-Loss Scale is fit-for-purpose for inclusion as a clinical trial endpoint in moderate-to-severe AD.

### Electronic supplementary material

Below is the link to the electronic supplementary material.

Supplementary Material 1

## Data Availability

The datasets generated during and/or analyzed during the current study are available from the corresponding author on reasonable request.
