# Supplementary material for: Sleep-loss related to itch in atopic dermatitis: assessing content validity and psychometric properties of a patient-reported sleep-loss rating scale
Source: J Patient Rep Outcomes. 2024 Jul 22;8:77. doi: 10.1186/s41687-024-00764-2 (PMC11263400; doi:10.1186/s41687-024-00764-2)
Supplement: Supplementary file 1 — Supplementary Material 1 [file 41687_2024_764_MOESM1_ESM.docx]

# Supplementary materials

## Conceptual saturation - Symptom and sleep impact in AD (total sample n=21)

| **Group 1**  **(n=4)** | **Group 2**  **(n=4)** | **Group 3**  **(n=4)** | | **Group 4**  **(n=4)** | | **Group 5**  **(n=4)** | |
| --- | --- | --- | --- | --- | --- | --- | --- |
| Burning  Itch  Rash  Redness  Skin cracked  Skin dry  Skin feels tight  Skin flaky  Skin irritation  Skin oozing or weeping  Skin peeling  Skin sensitive  Sleep: difficulty falling asleep  Sleep: difficulty staying asleep  Sleep: restless sleep  Sleep: tired next day  Sleep: unable to sleep  Soreness | Pain  Red spots  Stinging  Tingling |  |  | | -- | |  |
| 18 concepts | 4 concepts | 0 concepts | 0 concepts | | 0 concepts | |  |

## Participant-reported impact of AD (total sample n=21)

| **n ≥ 5 participants** | **n=4 participants** | **n=3 participants** | **n=2 participants** | **n=1 participant** |
| --- | --- | --- | --- | --- |
| Modifications to mitigate symptoms (n=16)  Scratch: need to (n=11)  Concentration (n=9)  Scratch: until bleeding (n=7)  Socializing (n=7)  Work (n=7)  Outdoor activities (n=6)  Scratch: in public (n=6)  Self-conscious (n=5)  Clothing choice (n=5)  Comments from others (n=5)  Daily activity interference (n=5)  Frustration (n=5) | Bleeding  Distraction   Embarrassment  Exercise  Annoyance  Sadness | Anxiety  Stress  Self-confidence  Scratch: redness from  Studying | Mess from shed skin  Range of motion  Conversation  Inconvenience  Infection | Open wounds Appearance Brushing teeth Doing dishes Dressing Family impact Handwashing Irritation (emotional) Isolation Mood School impact Using cleaning products |

## Measures

*Pruritis Numeric Rating Scale (PNRS)*

The Pruritus Numeric Rating scale (PNRS) is a novel scale that measures patient-reported itch severity. Patients assess their worst itch severity in the past 24 hours by choosing from its 11-point scale, with 0 indicating “No itch” and 10 indicating “Worst itch imaginable”.

*Patient-Oriented Eczema Measure (POEM)*

The Patient-Oriented Eczema Measure (POEM) is a patient- or observer-reported measure used to monitor disease activity in children and adults with AD. It consists of 7 items about some symptoms and impacts of AD, with each item scored on a 5-point scale ranging from “No Days” to “Every Day”. A total score out of 28 points is calculated based on item responses. The POEM has been evaluated for use in clinical practice and clinical trial settings [1].

*Dermatology Life Quality Index (DLQI)*

The Dermatology Life Quality Index (DLQI) is a patient-reported outcome (PRO) measure that aims to capture the quality-of-life impact of dermatological conditions on patients. It consists of 10 items about the impact of skin problems over the last week, with a 4-point response scale ranging from “Very much” to “Not at all”. The DLQI has been evaluated for use in clinical settings for various dermatological conditions, including AD [2].

*Hospital Anxiety and Depression Scale (HADS)*

The Hospital Anxiety and Depression Scale (HADS) is a PRO used to detect and evaluate levels of anxiety and depression [3]. It has been widely used, and has been evaluated in adult and adolescent populations [4, 5]. The HADS has subscales for depression and anxiety, each with 7 items. Each item is scored on a 4-point scale, ranging from 0 to 3, with 3 denoting the highest level of anxiety or depression. A total score of ≥8 out of 21 points on each subscale marks a significant level of anxiety or depressive symptoms.

*Global Assessment of Change for AD (GAC-AD)*

The Global Assessment of Change for AD (GAC-AD) is a single-item PRO. Patients were asked at the end of the clinical trial about their impression of the overall change in their AD.

## *Investigator’s Global Assessment (IGA)*

The Investigator’s Global Assessment (IGA) is an instrument used in clinical settings to rate the overall severity of the patient’s AD. IGA ratings are based on a 5-point scale, ranging from 0 (clear) to 4 (severe). The IGA must be conducted prior to conducting the EASI assessment.

*Body Surface Area (BSA)*

Body surface area (BSA) is a clinical tool for measuring the amount skin involvement in patients with AD. BSA is estimated based on sections of the body (e.g., head and neck, each arm, legs, and trunk). BSA can also be estimated based on patients’ “handprints”, with each palm-sized area reflecting approximately 1% of patient’s BSA [6].

*Eczema Area and Severity Index (EASI)*

The Eczema Area and Severity Index (EASI) is a clinician-reported tool used to evaluate the severity and extent of AD. A composite score from 0 to 72 is given, based on body area and severity sub-scores. The EASI has been evaluated as a comprehensive, fit-for-purpose tool for both adult and adolescent populations [7].

## Schedule of assessments

|  | Screening | Treatment Period | | | | | | | | | Follow-up/EOS | | | Early Termination |
| --- | --- | --- | --- | --- | --- | --- | --- | --- | --- | --- | --- | --- | --- | --- |
|  | V1 | V2 | V3 | V4 | V5 | V6 | V7 | V8 | V9 | V10 | V11 | V12 | V13 |  |
|  |  | W0 | W2 | W4 | W6 | W8 | W10 | W12 | W14 | W16 | W20 | W24 | W32 |  |
|  | -30 to -7 | D1 | D15 | D29 | D43 | D57 | D71 | D85 | D99 | D113 | D141 | D169 | D224 |  |
| IGA | **X** | **X** |  | **X** |  | **X** |  | **X** |  | **X** | **X** | **X** |  | **X** |
| EASI | **X** | **X** |  | **X** |  | **X** |  | **X** |  | **X** |  | **X** |  | **X** |
| BSA | **X** | **X** |  | **X** |  | **X** |  | **X** |  | **X** | **X** | **X** |  | **X** |
| Pruritus^a^ |  | **X** | **X** | **X** | **X** | **X** | **X** | **X** | **X** | **X** |  |  |  | **X** |
| Sleep-Loss^a^ |  | **X** | **X** | **X** | **X** | **X** | **X** | **X** | **X** | **X** |  |  |  | **X** |
| POEM |  | **X** |  |  |  |  |  |  |  | **X** |  |  |  | **X** |
| DLQI |  | **X** |  |  |  | **X** |  |  |  | **X** |  |  |  |  |
| HADS |  | **X** |  |  |  | **X** |  |  |  | **X** |  |  |  |  |
| GAC-AD |  |  |  |  |  |  |  |  |  | **X** |  |  |  |  |

**Legend:** Visit (V); Week (W); Day (D)

a: Pruritus NRS and Sleep-Loss Scale were completed daily on an electronic diary

## Effect sizes of the Sleep-Loss Scale


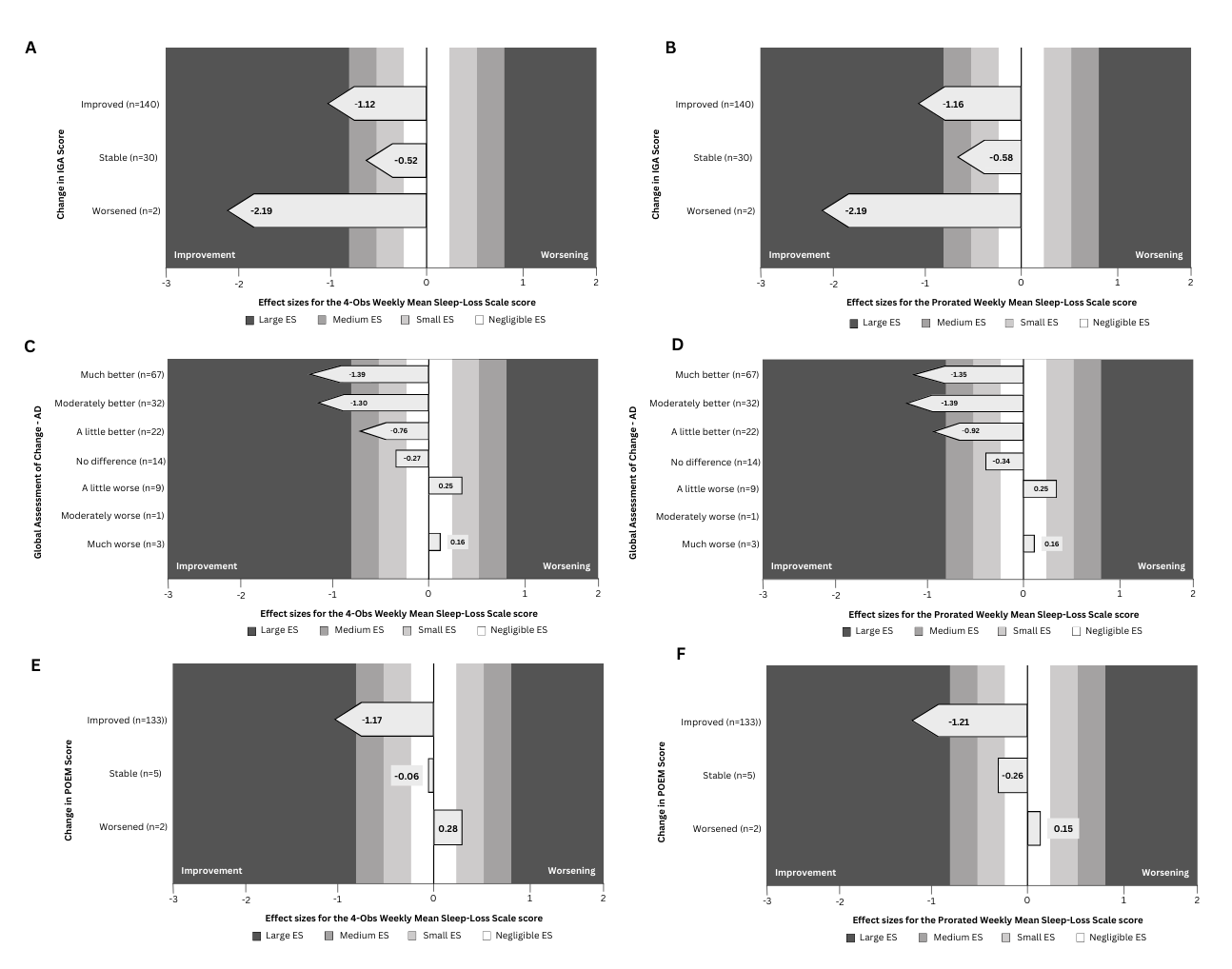


## References

1. Charman, C.R., A.J. Venn, and H.C. Williams, *The patient-oriented eczema measure: development and initial validation of a new tool for measuring atopic eczema severity from the patients’ perspective.* Archives of dermatology, 2004. **140**(12): p. 1513-1519.

2. Finlay, A.Y. and G. Khan, *Dermatology Life Quality Index (DLQI)—a simple practical measure for routine clinical use.* Clinical and experimental dermatology, 1994. **19**(3): p. 210-216.

3. Snaith, R.P., *The hospital anxiety and depression scale.* Health and quality of life outcomes, 2003. **1**(1): p. 1-4.

4. Herrmann, C., *International experiences with the Hospital Anxiety and Depression Scale-a review of validation data and clinical results.* Journal of psychosomatic research, 1997. **42**(1): p. 17-41.

5. White, D., et al., *Validation of the Hospital Anxiety and Depression Scale for use with adolescents.* The British Journal of Psychiatry, 1999. **175**(5): p. 452-454.

6. Ramsay, B. and C. Lawrence, *Measurement of involved surface area in patients with psoriasis.* British Journal of Dermatology, 1991. **124**(6): p. 565-570.

7. Hanifin, J., et al., *The eczema area and severity index (EASI): assessment of reliability in atopic dermatitis.* Experimental dermatology, 2001. **10**(1): p. 11-18.
